# Regulation of Shoot Apical Meristem and Axillary Meristem Development in Plants

**DOI:** 10.3390/ijms21082917

**Published:** 2020-04-21

**Authors:** Zhihui Xue, Liya Liu, Cui Zhang

**Affiliations:** 1 Key Laboratory of Plant Molecular Physiology, CAS Center for Excellence in Molecular Plant Sciences, Institute of Botany, Chinese Academy of Sciences, Beijing 100093, China; zhxue@ibcas.ac.cn (Z.X.); 18763820935@163.com (L.L.); 2 College of Life Sciences, University of Chinese Academy of Sciences, Beijing 100049, China

**Keywords:** shoot apical meristem, axillary meristem, transcription, plant hormones, epigenetics

## Abstract

Plants retain the ability to produce new organs throughout their life cycles. Continuous aboveground organogenesis is achieved by meristems, which are mainly organized, established, and maintained in the shoot apex and leaf axils. This paper will focus on reviewing the recent progress in understanding the regulation of shoot apical meristem and axillary meristem development. We discuss the genetics of plant meristems, the role of plant hormones and environmental factors in meristem development, and the impact of epigenetic factors on meristem organization and function.

## 1. Introduction

Plants are unique in their ability to continuously produce new organs throughout their life cycles. The process of continuous organogenesis depends on the activity of pluripotent cells. Some of these stem cells are located at the tips of shoot and root known as apical meristems. During embryogenesis, the shoot apical meristem (SAM) and root apical meristem (RAM) are established in the shoot apex and root apex, respectively. In monocots, such as rice and maize, the SAM is formed laterally, at the base of a single cotyledon [1]. In dicots, such as Arabidopsis, the SAM is established centrally, between two cotyledons. In Arabidopsis, during post-embryonic development, the SAM together with other meristems generates aboveground organs and the RAM contributes to underground architecture with the help of other meristems. Axillary meristems (AMs) form in the leaf axils to enable branching. During reproductive growth, the SAM transits into inflorescence meristem (IM). Then, the IM generates the floral meristem (FM), which produces floral organs [2]. 

SAM has dual functions of generating leaves, stems, and floral organs, as well as maintaining a pool of pluripotent cells in the center. The SAM contains three functional zones: The central zone (CZ), peripheral zone (PZ), and rib zone (RZ). The CZ is composed of slowly dividing pluripotent stem cells. The PZ, that surrounds the CZ, comprises rapidly dividing cells. Some progenies of the stem cells are displaced from the CZ into the PZ, where they give rise to lateral organs. The organizing center (OC), which is located within the rib meristem beneath the CZ, maintains the stem cell population [3]. In Arabidopsis, the SAM can also be classified into three clonally distinct layers of stem cells (L1–L3). The surface layer L1 forms the epidermis, L2 forms the subepidermal layer, and the L3 layer gives rise to the remaining inner corpus tissues of the shoot [4]. In Arabidopsis, AMs locate in leaf axils and share common features with the SAM such as generating leaves, stems, and maintaining pluripotent stem cells. The SAM and AMs together give rise to aboveground parts of the plant and determine plant architecture. In this review, we will summarize recent progress in the research on SAM and AM development and discuss how plant hormones, epigenetic factors, and environmental factors participate in the regulation of meristem development. All studies were conducted in Arabidopsis unless otherwise mentioned.

## 2. Establishment of the SAM

The SAM is established during embryogenesis. In Arabidopsis, the zygote divides asymmetrically to produce an apical cell and a basal cell. The smaller apical cell generates the embryo, while the larger basal cell forms the suspensor [5]. Asymmetric distribution of auxin mediated by the auxin efflux carrier PIN-FORMED (PIN) promotes the formation of an “apical-basal axis” in the embryo [6]. In the eight-cell embryo, the upper four cells give rise to the shoot meristem, whereas the lower cells generate the hypocotyl. A round of periclinal division gives rise to the 16-cell globular stage embryo. During the globular stage, the shoot meristem primordium is established. Establishment of the radial pattern of the embryo is a prerequisite for SAM formation and is regulated by two types of transcription factors (TFs): The class III homeodomain-leucine zipper (HD-ZIPIII) and KANADI (KAN) proteins [7]. The HD-ZIPIII proteins contain an HD-Zip domain, which is involved in DNA binding and protein dimerization [8]. The HD-ZIPIII transcription factor family consists of five members, namely REVOLUTA (REV), PHABULOSA (PHB), PHAVOLUTA (PHV), CORONA (CNA), and ATHB8 [9]. REV, PHB, and PHV begin to be expressed at the 16-cell globular stage, and their expression is confined to the central region of the embryo at the late globular embryo stage [7]. Consequently, the central region specifies the identity of the central cells. Meanwhile, KAN specifies peripheral identity in the developing embryo [10]. Bilateral symmetry is established at a later stage of embryo development in a process involving the interplay of auxin, the *SHOOT MERISTEMLESS* (*STM*) gene, and the *CUP SHAPED COTYLEDON* (*CUC*) genes [5]. *STM*, a member of the *KNOTTED-LIKE HOMEOBOX1* (*KNOX1*) gene family, encodes a class I KNOTTED-LIKE protein [11]. It maintains stem cell identity and regulates SAM cell division. The NAC domain transcription factors CUC1, CUC2, and CUC3 are involved in initiating the SAM [12]. CUC1 and CUC2 promote *STM* expression, and STM can both promote and repress *CUC1/2* gene expression, implying the existence of a positive and negative feedback loop between these genes [5,13,14]. Expression of the *CUC* genes is confined to a narrow strip, which is mediated by the auxin efflux carrier protein PIN1 and AUXIN RESPONSE FACTOR 5/MONOPTEROS (ARF5/MP) [12,15]. During the early stages of globular development, *STM* is expressed in the median region, which consists of a presumptive SAM region and a boundary region of cotyledon margins [16]. The cotyledons arise from the peripheral region, where *AINTEGUMENTA* (*ANT*) but not *STM* is expressed [17]. In summary, *HD-ZIPIII* genes, *STM,* and *CUCs* establish the radial and bilateral symmetry of the globular embryo, and thereafter, the SAM forms. 

## 3. Maintenance of Stem Cells in Meristems

**Figure 1:**
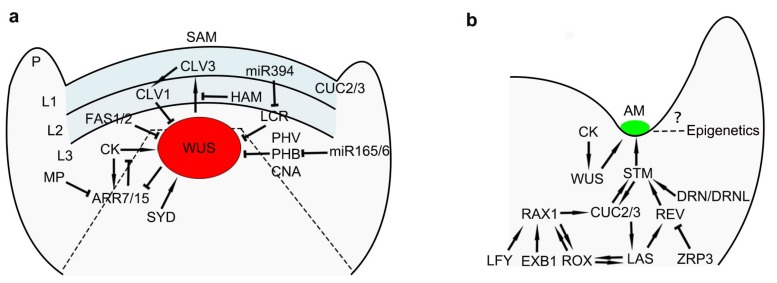
Conceptual models showing regulators in shoot apical meristem (SAM) and axillary meristems (AM) development. (**a**) A diagram illustrates the network in SAM regulation; P: Primordium. (**b**) A model shows factors involved in the regulation of AM development. Arrows and inhibition symbols indicate activation and repression, respectively.

The SAM harbors pluripotent stem cells. In Arabidopsis, the CLAVATA (CLV)-WUSCHEL (WUS) negative feedback loop is the central genetic mechanism that coordinates stem cell proliferation with differentiation in the meristem (Figure 1a) [18,19,20]. WUS, a homeodomain transcription factor, is required for regulation of meristem activity and stem cell maintenance [3,21]. Mutants for *WUS* fail to maintain stem cells, leading to the loss of the meristems and termination of organogenesis [22]. The *CLV* (*CLV1*, *CLV2*, and *CLV3*) genes play a role in promoting the progression of meristem cells toward organ initiation, which is opposite to that of *WUS*. Mutants for any of these genes lead to delayed organ initiation and accumulation of meristem cells [23,24,25]. *WUS* is initially expressed at the 16-cell embryo stage, when the SAM is not evident. The expression of *WUS* is restricted to the SAM OC [3]. Then, WUS migrates from the OC into the CZ region and activates *CLV3* expression exclusively in the outermost apical layer of the SAM by binding to *CLV3* promoter elements [3,25,26]. WUS-mediated *CLV3* activation is inhibited by the HAIRY MERISTEM (HAM) proteins [27]. *CLV3* encodes a putative ligand of the CLV1 receptor kinase, which is expressed throughout most of the SAM [28]. CLV3/CLV1 restrict *WUS* transcription to the OC via a receptor kinase-signaling cascade and thus limit the size of the OC [18,19,20,26,29]. This negative feedback loop coordinating stem cell proliferation and differentiation, appears to be conserved in diverse plant species [30,31]. It is possible that, *CLV* signaling and *WUS* are linked through the *CLV3* control of transcriptional regulators of *WUS*, such as S*TIMPY* (*STIP*), *SPLAYED* (*SYD*), *BARD1*, *OBERON1* (*OBE1*), and *OBERON2* (*OBE2*) [1]. SYD and BARD1 interact directly with the *WUS* promoter [32,33]; however, they have opposite functions. SYD is a SNF2 chromatin remodeling ATPase that promotes *WUS* transcription. In the *syd* mutant, the size of the meristems and transcriptional level of *WUS* are reduced. The BARD1 protein appears to be a repressor of *WUS*. In the *bard1* mutant, the meristem size is increased and *WUS* expression is upregulated. The Arabidopsis *OBE1* and *OBE2* genes are implicated in maintaining the expression of *CLV3* and *WUS* and maintaining stem cell activity [34]. 

The *KNOX* genes, including the Arabidopsis *STM*, have been proven to prevent the differentiation of stem cells. STM and WUS have distinct and complementary functions in the SAM. STM maintains the meristem cell fate and WUS specifies a subset of cells at the shoot apex as stem cells. In the *stm* mutant, the establishment and maintenance of the SAM are abolished. Thus, STM is vital for maintenance of meristematic activity. *STM* is expressed throughout the SAM [11] and prevents meristem cells from differentiating by promoting cytokinin synthesis [35]. In addition to *STM*, there are three other *KNOX* genes (*KNAT1*, *KNAT2,* and *KNAT6*) in Arabidopsis. They are localized at the shoot apex and function redundantly with *STM* [36]. These *KNOX* genes restrict the expression of the *ASYMMETRIC LEAVES1* (*AS1*) and *ASYMMETRIC LEAVES2* (*AS2*) genes to organ primordia, thus preventing ectopic organ initiation. *AS1* and *AS2* can in turn repress *KNOX* gene expression [37,38]. The interactions between *KNOX* transcription factors, which are expressed in the meristems, and *AS1*/*AS2*, which are expressed in the organ primordia, indicates that these genes are crucial for distinguishing stem cells and organ founder cells [36]. 

## 4. Initiation of AMs

**Figure 2:**
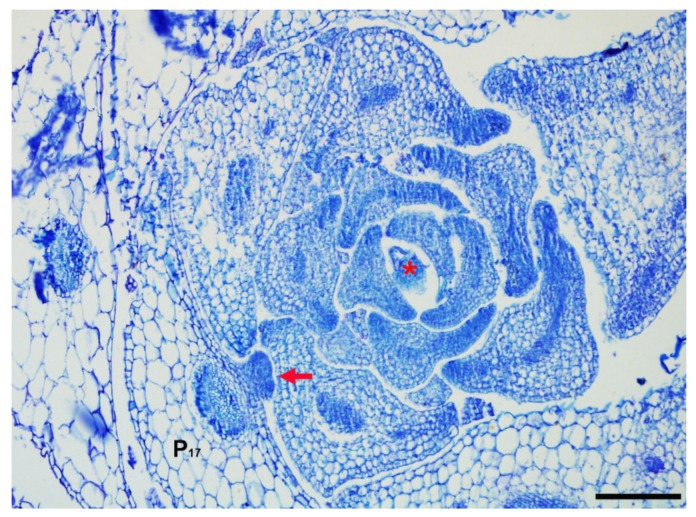
Axillary meristem and shoot apical meristem in Arabidopsis. A bump corresponding to an axillary meristem initiated in a mature leaf axil of P_17_ (the seventeenth earliest leaf primordium) is shown. The shoot apical meristem is located in the shoot apex. Arrow indicates axillary meristem and asterisk indicates shoot apical meristem. Bar = 100 µm.

Different from the SAM, AMs initiate at the base of adaxial side of leaves and develop at post-embryo stages. The microenvironment of leaf axils and developmental stages of leaves impact AM development. In seed plants, AMs enable lateral shoot branching. The development of a lateral shoot includes two steps: Initiation and outgrowth/dormancy. During AM initiation, a bump forms in the leaf axil and develops into an axillary bud (Figure 2). Owing to its subtle shape, AM development has been less studied. Several regulatory genes involved in AM initiation have been identified (Figure 1b), some of which participate in both SAM and AM development. For example, WUS and CLV3, which are required for SAM maintenance, are involved in AM initiation, and the meristem marker genes *STM* and *CUC* also participate in AM initiation. In weak *stm* mutants, which can survive to reproduce, there are significantly fewer axillary buds. The initiation of AMs requires a meristematic cell population continuously expressing *STM*, and the absence of *STM*-expressing cells abolishes bud formation [39]. *STM* expression is dynamic and is associated with two phases of stem cell division. During the first phase, in leaf axils younger than P_9_ (the ninth earliest leaf primordium), *STM* is expressed at a low level that is sufficient for stem cell competence but not for AM initiation [39]. It was recently reported that ARABIDOPSIS THALIANA HOMEOBOX GENE1 (ATH1) interacts with STM. The ATH1-STM heterodimer maintains *STM* expression and meristematic cell fate in the early leaf axil [40]. During the second phase, in leaf axils older than P_10_, *STM* expression is upregulated by REV and other transcription factors, and this propels bump formation. A transcriptional network contributes to the dynamic regulation of *STM*. *DORNRÖSCHEN* (*DRN*)/*ENHANCER OF SHOOT REGENERATION1* (*ESR1*) and its homolog *DORNRÖSCHEN-LIKE* (*DRNL*)*/ESR2*, which encode APETALA2 (AP2) type transcription factors, promoting AM initiation by activating *STM* [41]. In Arabidopsis mutants for these genes, AM initiation is compromised in the early rosette leaves [41,42]. During AM initiation, DRN/DRNL interact with REV to promote *STM* expression in leaves older than P_10_, while LITTLE ZIPPER3 (ZRP3), another REV-interacting protein interferes with the DRN/DRNL-REV complex, to prevent *STM* expression in leaf axils younger than P_9_ [41]. After the *STM*-induced gain of meristematic activity, de novo activation of the homeodomain transcription factor *WUS* by cytokinin signaling promotes AM initiation in the leaf axils around the P_13_ leaves [43]. *WUS* expression is also regulated by CLV3 in the AMs [44]. In addition, *CUC* genes are also expressed in both the SAM and AMs. In single and triple *CUC* gene mutants, AM formation is blocked to different extents due to the redundant functions of the *CUC* genes [45].

Different from the aforementioned genes, some transcription factors, including LATERAL SUPPRESSOR (LAS) and REGULATOR OF AXILLARY MERISTEMS (RAX), specifically regulate AM initiation. *LAS* encodes a GRAS family transcription factor, which is expressed in the leaf axil. Loss of function of *LAS* leads to severe defects in axillary bud formation [46]. *LAS* expression can be upregulated by *CUC2* [42]. The downstream target of *LAS* is *REV*, which encodes an HD-ZIP transcription factor as mentioned above, and is another gene involved in both SAM and AM initiation. REV binds to the *STM* promoter and directly upregulates *STM* expression in the leaf axil [39]. The *RAX* family genes, which encode R2R3 MYB transcription factors, are expressed in boundary regions and are specific regulators of AM initiation. *RAX1*, *RAX2,* and *RAX3* have redundant functions in promoting AM initiation. In *rax1 rax2 rax3* triple mutants, most axillary buds cannot form [47]. *RAX1* expression is regulated by a WRKY transcription factor EXCESSIVE BRANCHES1 (EXB1). Overexpression of *EXB1* leads to a bushy phenotype with excessive AM initiation [48]. LEAFY (LFY), which is required for the vegetative-reproductive stage transition, also upregulates *RAX1* expression to promote precocious AM development in rosette leaves [49]. *REGULATOR OF AXILLARY MERISTEM FORMATION* (*ROX*), which is regulated by *RAX* and *LAS*, is expressed in the leaf axils and promote AM initiation downstream of *RAX* and *LAS* [50]. As high throughput data and in-depth gene function data accumulate, the elaborate mechanisms underlying AM development will be unveiled.

## 5. Plant Hormones Regulate Meristem Development

Plant hormones, primarily auxin, cytokinin (CK), brassinosteroids (BRs), gibberellins (GA), and strigolactones (SLs) are signaling molecules that play important roles in a variety of physiological processes [51]. The interaction between cytokinin and auxin is required for the regulation of SAM, AM, and FM development [52,53,54,55]. In early embryogenesis, cytokinin and auxin control meristem formation. Stem cell maintenance requires high levels of cytokinin, which are achieved by repression of two negative regulators of cytokinin signaling, the *ARABIDOPSIS RESPONSE REGULATORS* (*ARR7* and *ARR15*). As previously mentioned, *WUS* defines the stem cell niche in the SAM. *WUS* is also required for stem cell definition during AM formation. Cytokinin signaling activates the expression of *WUS* in the leaf axil of P_13_, which is a requisite for AM initiation [43]. The *WUS* and auxin response factor ARF5/MP are both crucial for repression of *ARR7* and *ARR15* [55]. 

Auxin is required for organogenesis [56]. The boundary regions between the SAM and leaf primordia, show low auxin concentrations at an early stage before AM initiation, which was demonstrated using the auxin indicators DII and DR5 [57,58,59]. Ectopic application of an auxin signaling inhibitor resulted in supernumerary axillary buds indicating that a relatively low auxin level is required for boundary formation and the subsequent AM initiation. A high level of auxin in the boundary blocks the following *STM* expression in the leaf axil confirming that proper auxin is essential for boundary formation [39]. However, the role of auxin in AM initiation after boundary formation is not clear. The asymmetric distribution of auxin plays a vital role in the establishment of primordium, which is mediated by PIN proteins. Cytokinin can modulate the auxin distribution through transcriptional regulation of the *PIN* genes. Another factor required for auxin transcriptional response is MP/ARF5 [60]. MP regulates the polar localization of PIN1, which promotes the asymmetric distribution of auxin [61]. In addition, *PINOID* (*PID*), which encodes a serine-threonine protein kinase, regulates the polar subcellular localization of the PIN auxin efflux regulators [62]. In *pin1* and *pid* mutants, AMs are defective, again implying that auxin signals correlate with AM initiation.

Axillary buds can grow out into shoot branches or be inhibited in the leaf axil. The fate of an axillary bud is controlled by hormonal signals. Auxin, cytokinin, and strigolactones are important players during bud outgrowth. In pea, the export of auxin from axillary buds is a prerequisite for bud outgrowth, which is illustrated by the breaking of apical dominance [63,64,65]. Cytokinin plays a positive role late in AM development, and direct application of cytokinin promotes bud outgrowth in pea and *Jatropha curcas* [66,67,68]. Strigolactones, which act antagonistically to cytokinin in bud outgrowth, inhibit shoot branching [68,69]. 

## 6. Epigenetic Regulation of Meristem Development 

Epigenetic regulation, including DNA methylation, histone modifications, chromatin remodeling, and noncoding RNAs, plays important roles in plant development. The abovementioned regulators required for SAM maintenance, such as *WUS* and *STM*, are subject to epigenetic regulation. Chromatin remodelers regulate meristem activity by regulating *WUS* expression. Mutations in chromatin factors result in SAM dysfunction [70]. FASCIATA1 (FAS1) and FASCIATA 2 (FAS2) are two subunits of the CHROMATIN ASSEMBLY FACTOR 1 (CAF-1) complex. The *FAS1* and *FAS2* genes play a role in SAM organization. Mutations in *FAS1* and *FAS2* lead to aberrant SAMs with abnormal expression of *WUS* [71], thus leading to defects in shoot development. In addition, SYD and BRAHMA (BRM) which are SWITCH2/SNF2-related ATP-dependent chromatin remodeling factors, are expressed in meristems and involved in shoot development [72,73]. In the *syd* mutant, SAM formation is impaired and *WUS* expression is decreased. SYD regulates *WUS* expression by directly binding to the *WUS* promoter, indicating that *WUS* is a direct target of SYD [32]. In addition, the SYD binding protein BARD1 regulates SAM organization and maintenance by limiting *WUS* expression to the OC [33]. 

Histone modifications, such as methylation, acetylation, phosphorylation, and ubiquitination, can regulate gene expression. In Arabidopsis, *STM* is expressed exclusively in the SAM and silenced in lateral organ primordia. This expression pattern is considered to be important for SAM maintenance. Repression of *STM* in primordia is accomplished by a repressive mark, Histone 3 Lysine 27 trimethylation (H3K27m3), in the *STM* promoter. Polycomb Repressive Complex 2 (PRC2) establishes and maintains the H3K27m3 modification at target loci. In Arabidopsis, PRC2-like complexes consist of CURLY LEAF (CLF), SWINGER (SWN), EMBRYONIC FLOWER2 (EMF2), FERTILIZATION INDEPENDENT ENDOSPERM (FIE), and MULTICOPY SUPPRESSOR OF IRA1 (MSI1) [74]. In plants with mutations in these genes, H3K27 methylation was abolished at the *STM* locus, and *STM* was ectopically expressed [75]. PRC1 is required for maintaining the repressive marks established by PRC2, and there is evidence indicating that PRC1 also regulates *STM* expression [76,77].

Small RNAs are categorized into two major classes, miRNAs and siRNAs, based on the mode of biogenesis in the plant. Small RNAs play pleiotropic roles in plant development. They repress the target mRNAs mainly through post-transcriptional slicing and in part by translational inhibition. The RNA-induced silencing complex (RISC) consists of small RNAs and ARGONAUTE (AGO) proteins and is a key effector for target silencing [78]. Some *MIR* genes have been reported to be required for meristem development. For example, miR165/166 acting together with AGO1 regulate *HD-ZIPIII* genes, which are critical for stem cell maintenance, by cleaving their mRNAs [9,79,80]. ZWILLE/AGO10 (ZLL/AGO10), which is another important ARGONAUTE protein closely related to AGO1, also regulates *HD-ZIPIII* gene expression. Several studies have shown that ZLL/AGO10 and AGO1 have antagonistic functions with AGO10 functioning as a decoy protein. ZLL/AGO10 sequesters miR165/166, and also prevents the incorporation of miR165/166 into AGO1 [81], leading to HD-ZIPIII accumulation and SAM maintenance [82]. In addition, ZLL/AGO10 accelerates the degradation of miR165/166 by SMALL RNA DEGRADING NUCLEASES (SDNs) exonucleases, thereby favoring HD-ZIPIII accumulation [83]. 

The abovementioned STM-WUS-CLV pathway controls SAM maintenance. miRNAs also play vital roles in SAM maintenance by regulating targets in the STM-WUS-CLV pathway [84]. The decoy protein, ZLL/AGO10, is required for potentiating CLV3 and WUS activity in a non-cell-autonomous manner [85]. *WUS* expression is also indirectly regulated by miR394, which is synthesized in the L1 layer of the SAM. miR394 can diffuse from the L1 layer into the OC region, where it represses the F-box protein LEAF CURLING RESPONSIVENESS (LCR), a suppressor of *WUS*. Repression of LCR by miR394 is important for stem cell maintenance [29]. The NAC domain transcription factors CUC1, and CUC2 regulate organ boundary formation, and SAM and AM initiation, are targeted by miR164. In the *miR164* mutant, levels of *CUC* transcripts increased, which resulted in the formation of bud-like structures in the leaf margin [45,86]. *AP2* is a second class A gene that regulates FM termination. miR172 downregulates *AP2* expression through translation inhibition, which in turn represses stem cell fate and leads to the determination of FM identity [82,87,88,89]. Taken together, these studies indicate that miRNAs play an important role in SAM development; however, although *ago1* and *ago10* mutants display defective branches [90], much less is known about the roles of miRNAs in AM development (Figure 1b).

## 7. Environmental Factors Impact Meristem Development

Environmental signals, such as light, sugar, and nitrate modulate meristem development. These in vitro signals always interplay with plant hormones to regulate meristems. Light is not only a key energy source of plants but also an important player in shoot meristem development. In the dark, plants show skotomorphogenesis and SAM is dormant. In the light, plants display photomorphogenesis and meristems are activated [91]. Light activates *WUS* expression through CK signaling and also integrates with metabolic signals for stem cell activation in the SAM of Arabidopsis [92]. In tomato, light controls the initiation of leaves through auxin and CK [93]. The energy from light generates sugars and oxygen, which regulates meristem activity by converging on the TARGET OF RAPAMYCIN (TOR) kinase. TOR, which can respond to nutrient, energy, hormone, and environmental cues, is essential for metabolism, biogenesis, organ growth, and development transitions [94,95,96]. Auxin can substitute light to activate the TOR kinase in the presence of sucrose in Arabidopsis [97]. During SAM development, Arabidopsis plants adapt their organogenesis rate to the availability of nitrate in the soil. Cytokinin mediates this process by modulating *WUS* expression [98]. Oxygen is another important factor affecting meristem development. Recent studies confirmed that SAM develops in hypoxic conditions. Hypoxia protects LITTLE ZIPPER 2 (ZPR2), which is required for the activity of HD-ZIPIII transcription factors, thereby regulating shoot meristem activity [99]. Reactive oxygen species (ROS) play essentials roles in plants [100,101,102]. The mutation of an ATP-dependent mitochondrial protease, AtFTSH4, leads to the accumulation of oxidative stress in SAM at 30 °C and affects meristematic fate [103]. ROS contain the superoxide anion (O_2_^−^), hydrogen peroxide (H_2_O_2_), and the hydroxyl radical (OH). Different forms of ROS have different roles in stem cell regulation. The superoxide anion (O_2_^−^) activates *WUS* and maintains stem cell fate. In contrast, hydrogen peroxide (H_2_O_2_) promotes stem cell differentiation [104]. From these results, ROS are evidenced to be vital players in the maintenance of shoot stem cell niche [105]. 

How these environmental cues regulate AMs is still not clear. The outgrowth of lateral buds is regulated by environmental signals. Prolonged exposure to low temperatures can reactivate buds of the plants [106]. Another example is the inhibition of bud outgrowth when plants are subjected to far-red light, in which signal is mediated by the photoreceptor PHYTOCHROME B (PHYB). Consistent with this, in the sorghum and Arabidopsis *phyB* mutants, the plants have fewer branches [107,108]. There is evidence that nutrient deficiency also causes the inhibition of bud outgrowth [106]. In summary, because plants are immotile, environmental factors play rather important roles in regulating AM development. Although little is known about how the environment affects AM initiation, it is obvious that exogenous signals cooperate with plant hormones to influence bud growth and the whole plant architecture.

## 8. Future Perspectives

### 8.1. Cooperation of SAM and AM Development will Facilitate Systematic Design for High Yield Breeds in Agriculture

As environmental factors are unpredictable, modulation of plant development to shape fitness and achieve great yield in a variable environment is promising. Apical meristem and lateral branches are vital components of the aboveground architecture of the plant. A strong meristematic SAM and highly productive AMs are ideal traits pyramiding yield. The continuous activity of SAM was proposed to give rise to more lateral organs. A number of approaches have been adopted to screen diverse germplasms for prolonged meristem activity. However, longer periods of vegetative growth from the shoot apex are often accompanied by delayed maturity and an unintended reduction in yield [109]. Lateral shoots and inflorescence architecture determine seed number and yield, so alternative methods were used to increase the number of lateral branches and inflorescences through activation of AM initiation or outgrowth of lateral buds. However, few mutants with a larger number of AMs were obtained, potentially because of the tight regulation of AM initiation in leaf axils. In contrast, several bushy mutants showing increased bud outgrowth were identified, but grain yield was not significantly improved due to limitation of biomass in the whole plant and reduced growing density [110,111,112,113]. The relationship between the shoot apex and lateral branch is elucidated majorly by shoot apical dominance and in part by feedback from lateral organs. Different from canonical mutants with obvious phenotypes and a significant reduction in production, transformative modulation of gene expression at various levels (i.e., DNA, RNA, and protein) is prospective to provide more elite cultivars [114]. By taking advantage of recent genomic editing methods, the systematic design of innovative breeds through precise control of the SAM and AMs has become attainable [109].

### 8.2. Plant Hormones and Transcriptional Regulation Tailors Shoot

Plant hormones and transcription factors (TFs) play essential roles in plant development. A substantial number of genes important for meristem development, such as *STM* and *WUS* encode TFs. Transcriptional networks in SAMs centered on *STM*, *WUS,* and *CLVs* have been elucidated in detail [115,116]. More recently, a series of studies unveiled the transcriptional network underlying AM initiation, which features TFs specially expressed in AMs [117]. Altered expression of factors common to SAM and AM development always lead to defects in both types of meristems, with effects ranging from serious to lethal. In contrast, modulation of AM-specific genes allows specific control of AMs. Knockout mutants of AM-specific genes lead to fewer branches without defects in apical shoots [46,47]. Regarding yield, overexpression lines or knockout mutants of canonical meristem genes always display pleiotropic phenotypes along with a reduction or slight increase in yield, mostly because of the dominant function of TFs in plant development [9,118]. Inducible lines make it possible to control specific developmental stages, but these lines consume a lot of resources and application in the field seems impractical. Genomic editing enables precise control of gene expression through editing cis elements in the promoters or other noncoding regions at the nucleotide-scale. The ability to perform such precise editing has the potential to accelerate breeding for a high yield. Genome editing methods have already been applied in breeding of tomatoes and other crops [109,119,120].

### 8.3. Epigenetic Regulation Sculpts Shoot Architecture

Epigenetic regulation sculpts plant architecture [121]. Although the canonical epigenetic pathways have been uncovered, the function of epigenetic regulation in plant development needs to be elaborated. Genetic modification of DNA and changes in histone levels modify gene expression without altering DNA sequence. As outlined above, meristem modulators such as *WUS* and *STM* are modified at the epigenetic level to ensure appropriate expression during meristem formation. However, epigenetic modifications usually affect the whole genome. Tissue-specific modulation of epigenetic regulators is more feasible than constitutive expression. Genomic editing of DNA modification sites also provides an alternative means for construction of new transformants. miRNAs are a vital participant in plant development, and miRNA mutants display morphological phenotypes. Identifying miRNAs and analyzing their roles in AM initiation remains imperative. After being transcribed, targeted mRNAs, some of which are crucial for meristem development, are subjected to miRNA-mediated post-transcriptional gene silencing (PTGS) and translational inhibition. These regulatory pathways provide possibilities for modulating gene expression. In addition, the secondary structure of RNAs also affects PTGS and translation efficiency. As an increasing number of modifications are identified in either mRNAs or small RNAs, new methods for optimizing gene expression at the RNA level will shed light on the precise control of meristem development.

AMs located in leaf axils provide a good model for studying stem cell differentiation and meristem organization in a microenvironment. AMs are compact and are suitable for studying the short-distance movement of molecules. A further study of the movement of molecules among various cell types within AMs will aid the discovery of new mechanisms underlying plant development. 

## Abbreviations

SAM	Shoot apical meristem
RAM	Root apical meristem
AMs	Axillary meristems
IM	Inflorescence meristem
FM	Floral meristem
CZ	Central zone
PZ	Peripheral zone
RZ	rib zone
OC	Organizing center
L1	Layer 1
L2	Layer 2
L3	Layer 3
PIN	PIN-FORMED
HD-ZIPIII	Class III homeodomain-leucine zipper
KAN	KANADI
REV	REVOLUTA
PHB	PHABULOSA
PHV	PHAVOLUTA
CNA	CORONA
STM	SHOOT MERISTEMLESS
CUC	CUP SHAPED COTYLEDON
KNOX1	KNOTTED-LIKE HOMEOBOX1
ARF5	AUXIN RESPONSE FACTOR5
MP	MONOPTEROS
ANT	AINTEGUMENTA
CLV	CLAVATA
WUS	WUSCHEL
HAM	HAIRY MERISTEM
OBE1	OBERON1
OBE2	OBERON2
SYD	SPLAYED
STIP	STIMPY
BRAD1	BRCA1 associated RING domain 1
AS1	ASYMMETRIC LEAVES1
AS2	ASYMMETRIC LEAVES2
DRN	DORNRÖSCHEN
ESR1	ENHANCER OF SHOOT REGENERATION1
DRNL	DORNRÖSCHEN-LIKE
ZPR3	LITTLE ZIPPER3
LAS	LATERAL SUPPRESSOR
RAX	REGULATOR OF AXILLARY MERISTEMS
EXB1	EXCESSIVE BRANCHES1
LFY	LEAFY
ROX	REGULATOR OF AXILLARY MERISTEM FORMATION
CK	Cytokinin
BRs	Brassinosteroids
GA	Gibberellins
SLs	Strigolactones
ARR7	ARABIDOPSIS RESPONSE REGULATOR7
PID	PINOID
PHYB	PHYTOCHROME B
FAS1	FASCIATA1
FAS2	FASCIATA2
BRM	BRAHMA
H3K27m3	Histone 3 Lysine 27 trimethylation
PRC2	Polycomb Repressive complex 2
CLF	CURLY LEAF
SWN	SWINGER
EMF2	EMBRYONIC FLOWER2
FIE	FERTILIZATION INDEPENDENT ENDOSPERM
MSI1	MULTICOPY SUPPRESSOR OF IRA1
miRNAs	microRNAs
siRNAs	Small RNAs
RISC	RNA-induced silencing complex
AGO	ARGONAUTE
ZLL	ZWILLE
SDNs	SMALL RNA DEGRADING NUCLEASES
LCR	LEAF CURLING RESPONSIVENESS
AP2	APETALA2
TFs	Transcription factors
ATH1	ARABIDOPSIS THALIANA HOMEOBOX GENE1
